# Medicinal plants used by traditional medicine practitioners in treatment of alcohol-related disorders in Bushenyi District, southwestern Uganda

**DOI:** 10.3389/fphar.2024.1407104

**Published:** 2024-06-10

**Authors:** Samuel Maling, Jerome Kabakyenga, Charles Muchunguzi, Eunice Apio Olet, Mary Namaganda, Ivan Kahwa, Paul Erasmus Alele

**Affiliations:** ^1^ Department of Psychiatry, Faculty of Medicine, Mbarara University of Science and Technology, Mbarara, Uganda; ^2^ Department of Community Health, Mbarara University of Science and Technology, Mbarara, Uganda; ^3^ Department of Environmental and Livelihood Support Systems, Faculty of Interdisciplinary Studies, Mbarara University of Science and Technology, Mbarara, Uganda; ^4^ Department of Biology, Mbarara University of Science and Technology, Mbarara, Uganda; ^5^ Department of Plant Sciences, Microbiology & Biotechnology, School of Biosciences, College of Natural Sciences, Makerere University, Kampala, Uganda; ^6^ Department of Pharmacy, Faculty of Medicine, Pharm-Biotechnology and Traditional Medicine Centre of Excellence (ACEII), Mbarara University of Science and Technology, Mbarara, Uganda; ^7^ Department of Pharmacology and Therapeutics, Mbarara University of Science and Technology, Mbarara, Uganda

**Keywords:** ethnopharmacology, alcohol, alcohol-related disorders, medicinal plants, traditional medicine practitioners, Uganda

## Abstract

**Background:**

Alcohol-related disorders rank seventh among risk factors for morbidity and mortality globally, posing a significant public health burden. In Africa, including Uganda, there is limited availability and utilization of pharmacotherapies to treat alcohol-related disorders. This study documented medicinal plant species, plant parts used, and the methods of preparation and administration utilized by Traditional Medicine Practitioners (TMPs) in treating alcohol-related disorders in southwestern Uganda.

**Methods:**

A descriptive cross-sectional ethnopharmacological survey was conducted among TMPs within Bushenyi District, southwestern Uganda. Data was collected with key informant interviews using semi-structured questionnaires. The TMPs identified medicinal plants by local names. Plant specimens were collected and deposited at the Department of Biology, Faculty of Science, Mbarara University for identification and voucher numbers allocated. The plant scientific names and species were identified based on the International Plant Names Index. Plant species, family, life form, number of mentions, method of collection, preparation and administration were analyzed using descriptive statistics in Microsoft Excel. The survey data were utilized to compute Frequency of Citation, Relative Frequency of Citation, and Informant Consensus Factor.

**Results:**

We enrolled 50 traditional medicine practitioners aged between 34 and 98 years, with a mean age of 67. Approximately two-thirds were female (66%, 33/50), and mean experience in traditional healing was 31 years. The total number of plants identified were 25 belonging to 20 families. The most prevalent plant life form was herbs (36%) while grasses (4%), were the least. Leaves (48%) were the most utilized plant parts with the least utilized being the barks. The most prevalent method, adopted by approximately one-third of the TMPs, involved drying the plant material in the sun. The Informant Consensus Factor was 0.67.

**Conclusion:**

The study shows that the traditional medicine practitioners in Bushenyi district use a wide diversity of plants species to treat alcohol related disorders. The relatively high Informant Consensus Factor suggests a significant level of agreement among TMPs regarding the use of the identified plants. We recommend further investigations into phytochemistry, safety, efficacy, and mechanisms of action of the identified plants.

## Background

Globally, about 2.3 billion people are current drinkers of alcohol, and this is linked to immediate and long-term harmful effects on health and social and economic wellbeing (WHO, 2019a). Alcohol has a detrimental influence on 13 out of the 17 Sustainable Development Goals (SDGs), thus hindering progress towards health-related targets (Collin and Casswell, 2016). Alcohol abuse is directly linked to over 200 diseases and health conditions, including liver disorders, road traffic accidents, violence, cancer, cardiovascular diseases, mental disorders, suicides, tuberculosis, and HIV/AIDS (Bardach et al., 2019; Im et al., 2023). In 2016, alcohol misuse was globally ranked as the seventh leading risk factor of morbidity and mortality and was responsible for 5.1% of all Disability-Adjusted Life Years and 7.2% of all premature deaths (Griswold et al., 2018; WHO, 2019a). In the African region, harmful alcohol consumption contributes to 6.4% of all deaths and 4.7% of Disability Adjusted Life Years (Ferreira-Borges et al., 2016). Uganda is ranked among the highest consumers of alcohol in Africa, with a reported *per capita* consumption of 9.5 L, significantly surpassing the 6.3 L for the African region (WHO, 2019b). Furthermore, 28.8% of adult Ugandans aged 18 years and above are current alcohol drinkers, and 9.8% have an alcohol-related disorder (Kabwama et al., 2016). A high burden of alcohol has been demonstrated in studies in southwestern Uganda where it has been associated with risk of HIV infection and its treatment outcomes (Santos et al., 2014), intimate partner violence (Ohurira et al., 2023), risky behaviors (Bajunirwe et al., 2013; Nabifo et al., 2021), risk of mental illness (Bajunirwe et al., 2014) and injuries (Diamond et al., 2018).

While harmful alcohol consumption remains a significant public health concern, there is a limited availability and utilization of pharmacotherapies to complement psycho-social interventions (Carpenter et al., 2018). The three frequently employed pharmacotherapies (disulfiram, acamprosate, and naltrexone) for the treatment of alcohol-related disorders are of limited availability in resource-constrained settings and are associated with severe side effects the more serious ones being hepatotoxicity, renal impairment and peripheral neuropathy (Skinner et al., 2014). Exploring complementary and alternative pharmacotherapies such as herbal medicines with less side effects, and potential significant medical, social, and economic benefits, is therefore a viable option requiring research focus. The utilization of plant-based remedies for the treatment of alcohol-related disorders is documented in the literature (Singh et al., 2020). For example, traditional medicine approaches in the treatment of alcohol-related disorders using plants derivatives have been described by Xu et al. (2005), Lu et al. (2009) and Liu et al. (2011) in China, as well as by Corkery (2018a) in West Africa. Examples of medicinal plants studied internationally for the prevention and treatment of harmful alcohol drinking include *Kudzu* (*Pueraria montana* (Thunb.) Merr.) (Lukas et al., 2005; Lukas et al., 2013), *St. John’s wort* (*Hypericum perforatum* L.) (Rezvani et al., 1999), *ginseng* (*Panax ginseng* C. A. Mey.) (Carai et al., 2000; Lee et al., 2014), *Iboga* (*Tabernanthe iboga* Baill.) (Corkery, 2018b; Falkenhaug, 2023), Indian winter cherry (*Withania somnifera* (L.) Dunal) (Bansal and Banerjee, 2016) and milk thistle (*Silybum marianum* (L.) Gaertn.) (Tomczyk et al., 2012; Singh et al., 2020).

African traditional medicine (ATM) is defined by the World Health Organization as ‘the total knowledge, skills and practices based on the theories, beliefs, and indigenous cultural experiences, whether explicable or not, used in the maintenance of health, and diagnosing, preventing, or eliminating physical, mental or social diseases’ (WHO, 2013a). Herbal medicine, a major component of ATM, encompasses herbs, herbal materials, herbal preparations, and finished herbal products. These may consist of entire plants, plant parts such as leaves, bark, berries, flowers, and roots, and/or their extracts prepared with the intention for human therapeutic use or for other benefits in humans (WHO, 2001). African traditional medicine plays a significant role towards universal health coverage among people in sub-Saharan Africa, especially in rural areas where 60% of the population reside. ATM is often favored over modern medicine due to its cultural acceptance, affordability and accessibility, and also because the Traditional Medicine Practitioners are integral members of local communities.

According to the World Health Organization, the practitioner-to-population ratio for traditional medicine in Uganda stands at approximately 1:200–400. In comparison, there is just one Western-trained doctor for every 18,000 people (WHO, 2013b). This underscores the substantial contribution of traditional medicine to the country’s healthcare human resources. An estimated 200,000 TMPs exist in Uganda, serving a substantial portion of the population (Degonda and Scheidegger, 2012). It is further reported that 60%–79% of the Ugandan population turn to traditional medicine to meet their healthcare needs, including but not limited to diseases or conditions such as malaria, HIV, digestive and respiratory problems, toothaches, skin diseases, and childbirth complications, diabetes, snakebites and hypertension (Tugume et al., 2016; Opio et al., 2018; De, 2019; Gumisiriza et al., 2019; Asiimwe et al., 2021; Akwongo et al., 2022). While alcohol-related problems constitute a significant health concern in Uganda, the utilization of pharmacological treatments for its management remains limited. Research and anecdotal evidence suggest that TMPs in Uganda employ herbal remedies for addressing alcohol-related disorders (Maling et al., 2023). The objective of this study was to document the medicinal plant species, plant parts employed, and the methods of preparation and administration utilized by the TMPs in the treatment of alcohol-related disorders in the southwestern region of Uganda. The study also aimed at determining the level of agreement among Traditional Medicine Practitioners regarding the utilization of medicinal plants for treating alcohol-related disorders in southwestern Uganda.

## Methods

### Study area

**FIGURE 1:**
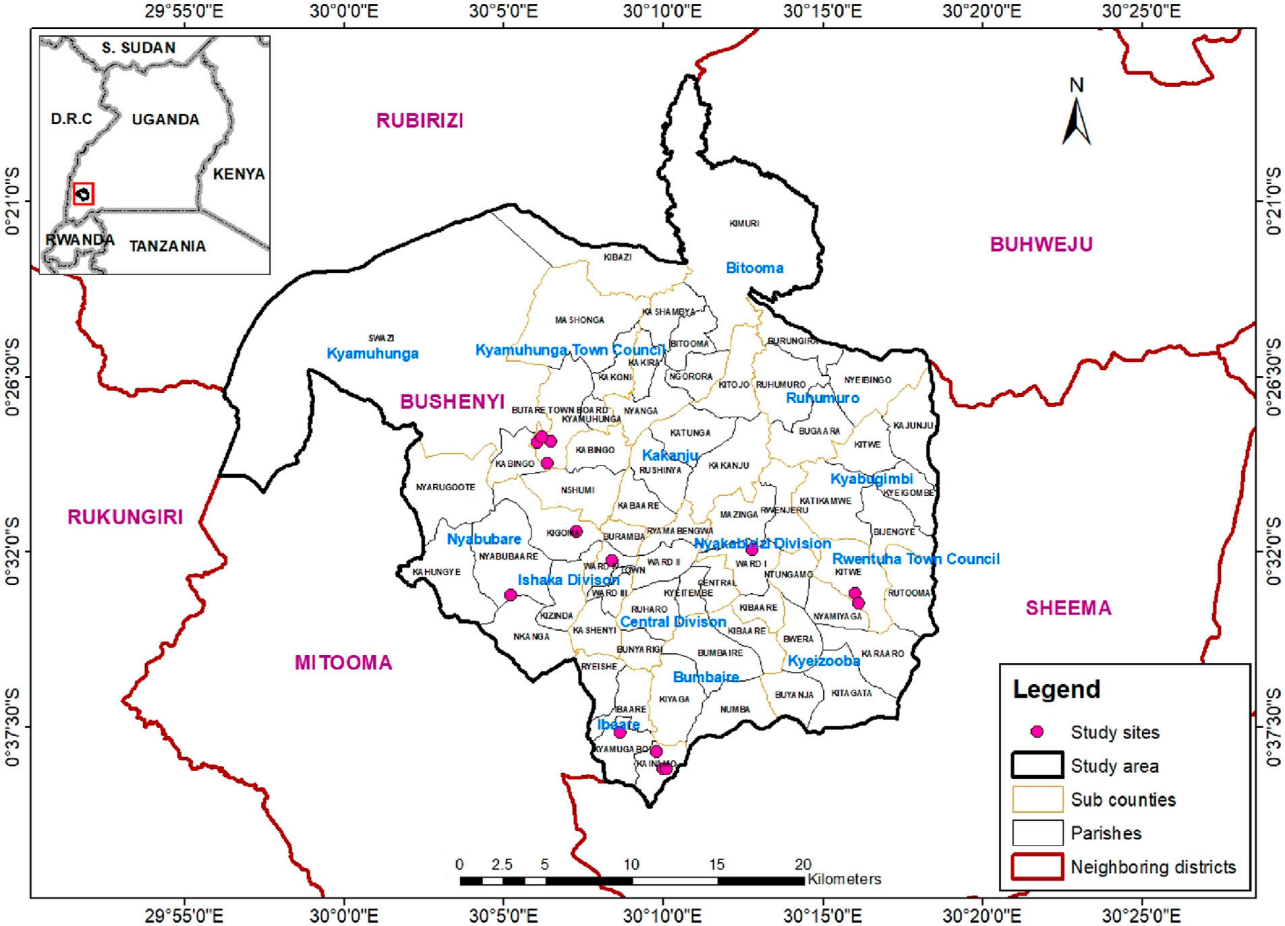
Map of Bushenyi District with inset showing location in Uganda (Map was created using ArcGIS version 10: 18/11/2023).

A cross-sectional ethnopharmacological survey was conducted in rural communities within Bushenyi District, located in southwestern Uganda (Figure 1 below). Data collection took place during the months of July and August 2020. Bushenyi District, located approximately 320 km southwest of the capital city Kampala, has a population of 246,100 people (UBOS, 2014), with the majority (73.8%) residing in rural areas. Geographically, the district lies at coordinates 0.4871° S and 30.2051° E, featuring low-lying plateaus intermingled with hilly terrain and receiving an annual rainfall of 1,500–2,000 mm. The area is adorned with tropical rainforests such as Kalinzu, Katsyoha-Kitomi, and Imaramagambo, that together cover 84 square kilometers (Bushenyi District Local Government, 2014), along with savannah woodlands and wetland vegetation. In addition, the district has fertile loamy soils with varying proportions of sand and clay. Most of the district’s population (87%) is engaged in subsistence agriculture, but are also involved in commercial tea, coffee and bananas cultivation. Other economic activities include fishing, stone quarrying, sand and mineral mining, tourism and lumbering. The main ethnic groups in the district are the Banyankole, who are the majority, along with the Bakiga and Baganda. The main local language spoken is Runyanore-Rukiga. We chose Bushenyi district as our study area due to the presence of an organized group of traditional healers, the Bushenyi Medical and Traditional Healers Association (BUMETHA). Founded in 1988, BUMETHA actively fosters collaboration between modern and traditional medicine, including in research, some of which has been published (Tumwesigye, 1996; Catherine et al., 2020; Primah, 2023).

#### Study participants

We recruited traditional medicine practitioners with experience in treating patients with alcohol drinking problems using medicinal plants and residing within the study area. To be included in the study, the traditional medicine practitioner had to be a resident in the study area and have experience in managing alcohol related problems using medicinal plants. The traditional medicine practitioners who were unwilling to share the plants they used were excluded from the study.

#### Sample size and sampling

A sample size of 50 TMPs was determined based on a precedent from a similar study conducted in Uganda (Ssenku et al., 2022) as well as sample size estimation techniques for qualitative studies described by Morse (Morse, 2015). In this guide by Morse, a sample size of 30–60 for qualitative ethnographic studies is recommended. We therefore estimated our sample size *a priori* at 50 basing on this guide and a similar study done in Uganda. We purposively selected the TMPs who treat alcohol disorders using the snowball technique (Parker et al., 2019). The process commenced with the chairman of their association, who provided the initial contacts.

#### Data collection process

Data was collected using key informant interview technique and semi-structured interview guide and questionnaire developed by the research team. The interview guide collected data on socio-demographic characteristics, physical address of traditional medicine practitioner, plant identification using local names, plant parts used and collection, methods of preparation and administration.

The key informant interviews were conducted by three research assistants all conversant with the local culture and fluent in the local language (Runyankore-Rukiga) as well as English. All the three research assistants had experience of working with traditional healers in research and collaboration projects in southwestern Uganda. In addition, they had a health background, two in nursing and mental health and the other in public health. Details of the questionnaire and interview guide are shown in Table 1 below.

**TABLE 1:** Questionnaire and interview guide.

Interview area	Guiding questions
Socio-demographic characteristics	Age, gender, religion, main occupation, other occupations, marital status, highest education level, duration of practice in traditional medicine, telephone contact
Physical address of traditional medicine practitioner	Please share
	Name of village where you reside
	Name of your parish
	Name of your subcounty
	Name of your district
Plant identification	Please share the local name of the plant that you use to treat people with drinking problems
	*Probe if there are distinguishing aspects of the plant*
Plant parts used and collection	Please share what part or parts of the plant that you use
	*Probe detailed process of collection step by step*
	*Probe how the parts are collected, season if flowers or seeds*
Preparation	Please describe how you prepare the plant material to make the remedy that you use to treat people with alcohol problems
	*Probe step by step of detailed process*
Administration	Please share how you administer the remedy
	*Probe how much and how often*
Plant collection during field trip	Date of plant collection
	Conservation status of plant: *probe if plant is common, rare, very rare*
	Life form of the plant
	Village, parish and sub-county where plant is located
	GPS coordinates and altitude where plant is located
Scientific identification at herbarium	Voucher number
	Scientific name
	Species
	Family

#### Plant identification

The research team and the traditional medicine practitioners made field trips to where the plants grew. The traditional medicine practitioners identified the medicinal plants they use to treat alcohol by local names. Voucher specimens of the plants mentioned by the TMPs were then collected using a procedure described by Martins and others (Martin, 2010) and taken to a botanist at the Department of Biology, Faculty of Science, Mbarara University of Science and Technology where voucher numbers were assigned. The scientific names of the plants and species were identified based on the International Plant Names Index (Ipni, 2012). The voucher specimens were preserved at the Department of Biology of Faculty of Science, Mbarara University.

#### Quantitative ethnobotany

Data obtained from the survey were used to calculate the following ethnobotanical indices: Frequency of Citation (FC) (Tardío and Pardo-de-Santayana, 2008; Faruque et al., 2018), Relative Frequency of Citation (RFC) (Tardío and Pardo-de-Santayana, 2008; Faruque et al., 2018) and the Informant Consensus Factor (ICF) (Tardío and Pardo-de-Santayana, 2008; Faruque et al., 2018). The indices were calculated as follows (Faruque et al., 2018):

Frequency of Citation (FC)

$$\text{FC}=\frac{\left(\text{Number}\;\text{of}\;\text{mentions}\;\text{of}\;a\;\text{particular}\;\text{plant}\;\text{species}\right)}{\left(\text{Total}\;\text{number}\;\text{of}\;\text{mentions}\;\text{of}\;\text{all}\;\text{species}\;\right)\;}\times 100$$

Relative Frequency of Citation (RFC)

$$\text{The RFC}=\frac{\text{Number}\;\text{of}\;\text{informants}\;\text{who}\;\text{mentioned}\;\text{each}\;\text{plant}\;\text{species}\;}{\text{Total}\;\text{number}\;\text{of}\;\text{informants}\;\text{in}\;\text{the}\;\text{study}}$$

The RFC index ranged from “0” when nobody referred to a plant as useful to “1” when all informants referred to a plant as useful.

### The informant consensus factor

The Informant Consensus Factor (ICF) is a measure used in ethnopharmacological studies to quantify the level of agreement among traditional medicine practitioners regarding the use of specific plants for a particular ailment category, which is alcohol in this study. The ICF was calculated in this study using the formula (Oryema et al., 2021): ICF = $\left(N_{\text{ur}}{\;-\;N}_{t}{)/\;(N}_{\text{ur}\;}-1\right)$ where N_ur_ is the number of use reports or mentions and N_t_ is the number of species used.

#### Data analysis

Plant species, family, life form, number of mentions by TMPs, method of collection, preparation and administration, Frequency of mention and Relative Frequency of mention were analyzed using simple descriptive statistics in Microsoft Excel 2019 (Frye, 2018) and summarized in frequency tables and figures.

#### Ethical considerations

All study participants provided individual written informed consent. For those participants who lacked formal education or could not read and write, the consent form was read aloud to them verbatim, and they expressed their consent by providing a thumbprint. Throughout the research process, the research team maintained a respectful and considerate relationship with local communities and authorities. Additionally, the team took precautions to ensure that the locations from which the plants were collected held no spiritual significance that could potentially conflict with the research objectives. Plant samples were meticulously collected with a strong focus on conservation, making every effort to avoid depletion of plant populations. In the case of cultivated plants, specific permission was obtained prior to collection. The research was reviewed and approved by the Mbarara University Research Ethics Committee (#39/02-20).

## Results

### Sociodemographic characteristics of the traditional medicine practitioners

Fifty traditional medicine practitioners were enrolled into the study. The ages of the TMPs ranged from 34 to 98 years, with a mean age of 67. The 25th percentile age was 59 years, indicating that three-quarters of the traditional healers were older than 59 years. The mean experience of the TMPs was 31 years, while the 50th percentile years of experience in traditional healing was 30, indicating that half of the healers had more than 30 years of experience in traditional healing. Approximately two-thirds of the TMPs (33/50, 66%) were female. The majority of participants (42/50, 84%) had either not received formal education or had completed only primary education. Nearly all (46/50, 92%) were engaged in additional occupations alongside their traditional medicine practices, primarily in peasant farming and informal businesses. Only four of the participants exclusively practised traditional medicine without any other occupation. Details of the socio-demographic characteristics are in Table 2 below.

**TABLE 2:** Sociodemographic characteristics of traditional medicine practitioners managing alcohol-related disorders in Bushenyi District.

Socio-demographic	Number (*n* = 50)	%
Sex		
Female	33	66.0
Male	17	34.0
Education		
No formal education	23	46.0
Primary	19	38.0
Secondary	4	8.0
Tertiary	4	8.0
Marital status		
Married	28	56.0
Widowed	21	42.0
Single	1	2.0
Religion		
Protestant	16	32.0
Catholic	24	48.0
Moslem	8	16.0
Born again	2	4.0
Other main occupation		
Farming	28	56.0
Business	5	10.0
Formal employment	5	10.0
Traditional birth attendant	2	4.0
Crafts	1	2.0
Retired	1	2.0
None	8	16.0
Tribe		
Munyankole	40	80.0
Muganda	3	6.0
Other tribes	7	10.0

### Plant identification

**FIGURE 2:**
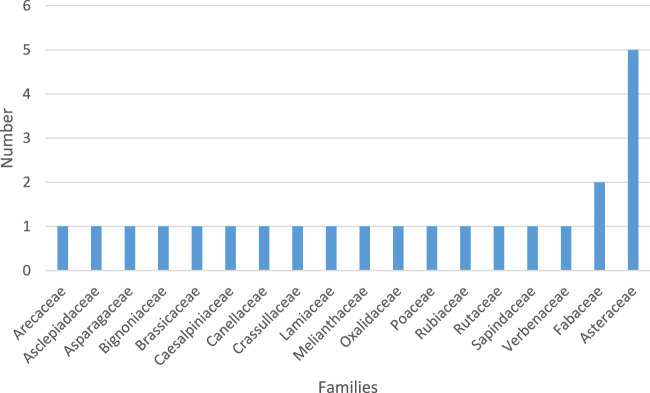
Families of plants used by Traditional Medicine Practitioners for treating alcohol-related disorders.

The total number of plant species identified were 25 belonging to 20 families (Figure 2 below). Asteraceae was the predominant family.

The four most commonly used ethnomedicinal plant species were *Dracaena fragrans* (L.) Ker Gawl., *Coffea canephora* Pierre ex A. Froehner, *Saccharum officinarum* L. *and Melanthera scandens* (Schumach. and Thonn.) Brenan (Details in Table 3 below).

**TABLE 3:** Medicinal plant species used by traditional medicine practitioners in the treatment of alcohol-related problems in Bushenyi district.

S/N	Scientific name and voucher number	Local name (runyankore)	Family	Life form	Part (s) used	Frequency of citation (FC)	Conservation status	Relative frequency of citation (RFC)
1	*Dracaena fragrans* (L.) Ker Gawl., SM2	Omugorora	Asparagaceae	Shrub	Flowers	29.7	Common	0.44
					Seeds			
					Leaves			
2	*Coffea canephora* Pierre ex A. Froehner, SM1	Coffee/omubarama/Omwani	Rubiaceae	Tree	Flowers	25.7	Common	0.38
					Leaves			
3	*Saccharum officinarum* L., SM7	Sugarcane/Ekikwaijo	Poaceae	Grass	Flowers	6.8	Common	0.10
4	*Melanthera scandens* (Schumach. and Thonn.) Roberty, SM18	Ekarwe	Asteraceae	Herb	Leaves	4.1	Common	0.06
5	*Bersama abyssinica* Fresen., SM4	Omukaka	Melianthaceae	Herb	Leaves	2.7	Common	0.04
6	*Erythrina abyssinica* Lam., SM12	Ekiko	Fabaceae	Shrub	Flowers	2.7	Common	0.04
7	*Kigelia africana* (Lam.) Benth., SM6	Omusha/omwisya	Bignoniaceae	Shrub	Leaves	2.7	Rare	0.04
					Flowers			
8	*Solanecio cydoniifolius* (O. Hoffm.) C. Jeffrey, SM11	Irarira	Asteraceae	Shrub	Roots	2.7	Rare	0.04
9	*Solanecio mannii* (Hook. fil.) C. Jeffrey, SM22	Engango	Asteraceae	Tree	Leaves	2.7	Common	0.04
10	*Biophytum abyssinicum* Steud. ex A. Rich., SM9	Irango	Oxalidaceae	Herb	Root	1.4	Rare	0.02
11	*Brassica oleracea* L. var. *capitata* f. *rubra* DC, SM3	Purple Cabbage head	Brassicaceae	Herb	Leaves	1.4	Common	0.02
12	*Clerodendrum myricoides* R. Br., SM8	Omukuzanyana	Lamiaceae	Shrub	Flowers	1.4	Rare	0.02
					Fruits			
13	*Dodonaea viscosa* (L.) Jacq., SM25	Omusambya	Sapindaceae	Herb	Leaves	1.4	Common	0.02
14	*Gomphocarpus physocarpus* E. Mey., SM21	Kashaho/Kanyamate	Asclepiadaceae	Herb	Leaves	1.4	Rare	0.02
15	*Kalanchoe pinnata* (Lam.) Pers., SM16	Ereka	Crassulaceae	Herb	Roots	1.4	Common	0.02
16	*Lantana trifolia* L., SM15	Omuhukye	Verbenaceae	Shrub	Leaves	1.4	Common	0.02
17	*Momordica foetida* Schumach., SM14	Ebombo	Cucurbitaceae	Vine	Leaves	1.4	Common	0.02
18	*Phoenix reclinata* Jacq., SM23	Ekikindo	Arecaceae	Tree	Leaves	1.4	Common	0.02
19	*Rumex usambarensis* Dammer ex Peter, SM5	Omuka	Polygonaceae	Shrub	Leaves	1.4	Common	0.02
20	*Senna didymobotrya* (Fresen.) H. S. Irwin and Barneby, SM17	Omugabagaba	Caesalpiniaceae	Tree	Leaves	1.4	Common	0.02
21	*Tephrosia nana* Kotschy ex Schweinf., SM24	Abati muruku	Fabaceae	Tree	Leaves	1.4	Common	0.02
22	*Vernonia brachycalyx* O. Hoffm., SM19	Omuhe	Asteraceae	Herb	Roots	1.4	Common	0.02
23	*Vernonia lasiopus* O. Hoffm., SM13	Omujuma	Asteraceae	Herb	Leaves	1.4	Common	0.02
24	*Warburgia ugandensis* Sprague, SM10	Omwiha	Canellaceae	Tree	Bark	1.4	Rare	0.02
25	*Zanthoxylum gilletii* (De Wild.) P. G. Waterman, SM20	Omuremankobe/Omurema	Rutaceae	Tree	Bark	1.4	Rare	0.02

### Life forms of the plants used by traditional medicine practitioners in Bushenyi District to manage people with alcohol-related disorders

**FIGURE 3:**
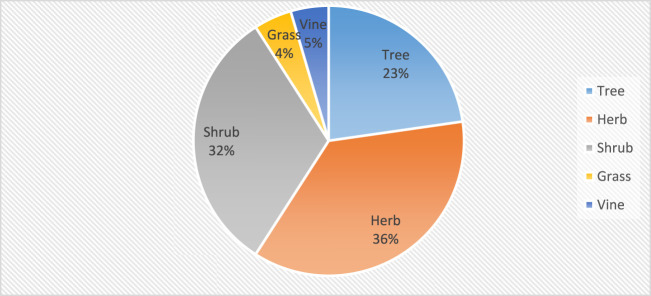
Life forms of plants used by traditional medicine practitioners to treat alcohol-related disorders.

The most prevalent life form of plants employed was the herbs (36%), while grasses (4%), were the least frequently utilized, as depicted in Figure 3 below.

### Plant parts used by traditional medicine practitioners in Bushenyi District to manage people with alcohol-related disorders

**FIGURE 4:**
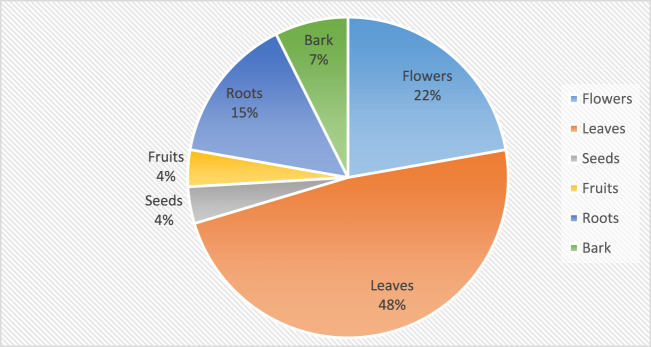
Plant parts used by traditional medicine practitioners in the treatment of alcohol-related disorders.

Leaves (48%) were the most utilized plant parts, while fruits and seeds were the least used, each contributing only 4%, as depicted in Figure 4 below.

### Methods used to prepare and administer plant materials

**FIGURE 5:**
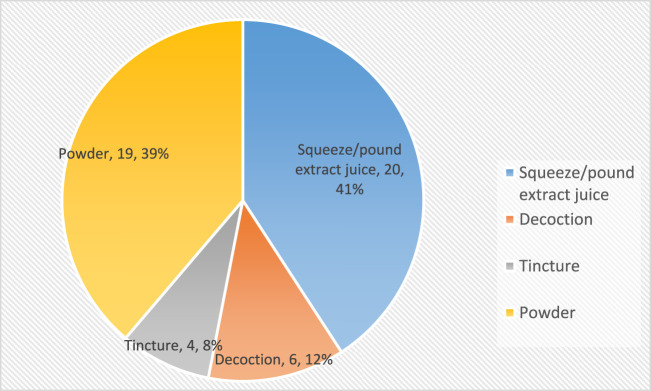
Methods used by traditional medicine practitioners to prepare medicinal plants treating alcohol-related disorders.

Among the 50 Traditional Medicine Practitioners interviewed, 40% exclusively utilized a single plant, 38% combined two plants, and 19% mixed three or four plants in the preparation of herbal remedies for alcohol. The traditional healers utilized four methods to prepare the medicinal plant remedies. The decoction method involved the prolonged boiling of plant parts, such as roots, bark, or seeds, in water to extract medicinal compounds. Tinctures were made by immersing plant parts in alcohol or a mixture of alcohol and water. Additionally, plant materials were dried and processed into powders for application. Finally, certain healers employed the technique of pounding or squeezing fresh plant parts to extract beneficial juices. The most prevalent techniques employed by the Traditional Medicine Practitioners for preparing medicinal plant remedies were squeezing or pounding plant parts to extract juice, utilized by 41% of the healers, and the least was tincturing used by only 8% of the healers. In terms of administering the powder or juice, all the TMPs, with the exception of one, mixed 1–2 spoonfuls of the powder or juice with alcohol, which was then given to the patient for consumption. The sole exception to this practice involved a TMP who combined the powder with a ripe banana, forming a paste that was given to the patient for ingestion. Figure 5 below shows the methods used by the healers in the preparation of the medicinal plant parts.

### Quantitative ethnobotany

In this survey, the Relative Frequency of Citation (RFC) ranged between 0.02 and 0.44. Based on this RFC data, the four most commonly used ethnomedicinal plant species were *D. fragrans* (0.44), *C. canephora* (0.40), *S. officinarum* (0.1) and *M. scandens* (0.06).

We calculated the Informant Consensus Factor (ICF) using the following formula: ICF = (N_ur_ - N_t_)/(N_ur_ −1) where N_ur_ is the number of use reports or mentions and N_t_ is the number of species used. In this study, N_ur_ was 74, and N_t_ was 25. Therefore ICF = $\left(74\;–\;25\right)/\left(74-1\right)$ = 0.67

## Discussion

Ethnopharmacological surveys play a crucial role in enabling scientists to identify plants with potential medicinal properties, that can be further integrated into healthcare and support healthcare provision. In this study, we undertook an ethnobotanical survey to document both the plants as well as associated practices employed by TMPs for treating alcohol-related disorders in Bushenyi District, southwestern Uganda.

### Participant characteristics

The observation in this study that the majority of TMPs were women, and predominantly older, aligns with findings in various regions of Uganda (Akwongo et al., 2022; Ssenku et al., 2022). However, it is divergent from other studies that reported a predominant male presence (Mongalo and Raletsena, 2022; Tabuti et al., 2023). The higher representation of female traditional medicine practitioners in comparison to their male counterparts can be attributed to women taking on more roles as primary healthcare providers for their families, in line with societal traditions. Consequently, the responsibility of treating individuals with alcohol-related disorders often falls upon women. Furthermore, the notable representation of older individuals among the participants in this survey corresponds with observations in other studies conducted in Uganda and the wider region (Mukazayire et al., 2011; Kamagaju et al., 2013). Older individuals in society often possess extensive knowledge of medicinal plants and their uses, attributed to their prolonged and direct interaction with plant resources.

### Diversity of medicinal plants

Our observation that the most utilized plant species employed by traditional medicine practitioners belonged to the Asteraceae family aligns with other studies in Uganda and other African regions which acknowledged its significant medicinal value (Hamill et al., 2000; Tefera and Kim, 2019; Ssenku et al., 2022). These plants are widely distributed in Uganda and the chances of TMPs using them for medicinal purposes are high (Mbatudde et al., 2007). The predominant use of these families may also be due the fact that most plant species in this family are mainly herbs and shrubs, so grow quickly and available throughout the year. The four most commonly used ethnomedicinal plant species were *D. fragrans, C. canephora, S. officinarum and M. scandens.* In contrast, only one plant, *Dracaena steudneri Engl*., belonging to the Dracaenaceae family, was identified for its use in treating alcohol-related disorders in an ethnobotanical study carried out in communities surrounding Mabira Forest in Central Uganda (Tugume et al., 2016). This finding confirms the use of medicinal plants in the traditional treatment of alcohol-related disorders across different regions of Uganda.

### Plant life forms and parts used

Comparable to other studies, this survey found that herbs were the most predominant plant life form utilized by the TMPs for treating alcohol-related disorders, with shrubs and trees following in frequency. The common use of herbs, trees and shrubs has been reported in most studies in Uganda and elsewhere (Nankaya et al., 2019a; Ojelel et al., 2019; Girma et al., 2022). Their widespread utilization can be attributed to their abundance in gardens, bushes, and forests, as well as their ease of collection. The high utilization of leaves could be attributed to the ease with which they can be collected in large quantities compared to other plant parts (Nankaya et al., 2019b). Studies reporting the preference for leaves by traditional medicine practitioners suggest that it is driven by their abundance and the high concentrations of compounds with diverse medicinal properties found within leaves (Nankaya et al., 2019b). Similar findings have been described in Uganda (Hamilton, 2004; Kamatenesi-Mugisha and Oryem-Origa, 2007; Ssegawa and Kasenene, 2007; Okot et al., 2020; Mongalo and Raletsena, 2022) and other countries in Africa (Mongalo and Raletsena, 2022; Feyisa et al., 2023). The prevalent utilization of leaves, roots, flowers, and barks in herbal medicine preparations is a widespread practice across numerous communities in Uganda, as documented in central Uganda (Kamatenesi et al., 2011), southern Uganda (Ssegawa and Kasenene, 2007), western Uganda (Asiimwe et al., 2013), communities surrounding Kibale National Park (Namukobe et al., 2011), and Mpigi in central Uganda (Schultz et al., 2020). Similar practices are also reported in other countries, such as Tanzania (Amri and Kisangau, 2012), Ethiopia (Moges and Moges, 2019) and Italy (Petelka et al., 2020).

### Homogeneity of use

A relatively high ICF observed in this study is comparable to other studies. High ICF were noted for plants used by traditional medicine practitioners in treating breast cancer (Lutoti et al., 2023), malaria (Adia et al., 2014) and other ailments (Ssenku et al., 2022). Other studies in Uganda have reported even higher ICF levels of up to 0.9 (36). The high ICF signifies high levels in agreement among the healers on the plants used to treat alcohol related disorders. The difference in informant consensus factor in this study compared to others may stem from variations in ecological diversity and the extent of knowledge sharing within the study areas.

### Preparation and administration of remedies

The methods employed by the TMPs in preparation of medicinal plant remedies in our study aligns with the findings of another study conducted in southwestern Uganda (Gumisiriza et al., 2019) which highlighted crushing/pounding then mixing in water as a common method for preparing herbal remedies. Powders are preferred primarily for their prolonged shelf life, proving especially beneficial when utilizing seasonal plant parts like flowers and fruits. In contrast, infusions are consumed within a day, while decoctions are stored in tightly sealed containers for at least 1 week at room temperature. However, this finding contrasts with other research conducted in Uganda and the broader East African and African regions, where decoction is frequently identified as the dominant method of herbal preparation (Alebie et al., 2017; Gumisiriza et al., 2023). Decoctions may be preferred when the part of the plant used is available all year round such as leaves and bark. The widespread use of water as a solvent is attributable to its ready availability and its ability to dissolve a large number of metabolites. This property allows for the swift extraction of active ingredients during the preparation of herbal remedies (Alebie et al., 2017). An interesting observation by healers of mixing the prepared powder or tincture into alcohol is contradictory. The healers may see this as the easiest method to administer the remedy.

## Conclusion, limitations and recommendations

This ethnobotanical survey has shown that the traditional medicine practitioners in Bushenyi district use a wide diversity of plants species to treat alcohol related disorders. The relatively high Informant Consensus Factor suggests a significant level of agreement among TMPs regarding the use of the identified plants. A limitation of this study may be bias among the TMPs who may have withheld names of some of the plants they use. This was mitigated by recruiting a bigger number of the TMPs into the study. We recommend investigations into phytochemistry, potential safety, efficacy, and mechanisms of action of the identified plants, especially those with a high frequency of citation such as *D. fragrans*. Additionally, conducting clinical trials to evaluate the effectiveness of the identified plants would contribute valuable evidence to guide healthcare practices in alcohol treatment. This will be novel in the African region regarding plant remedies for alcohol treatment.

## Data Availability

The raw data supporting the conclusion of this article will be made available by the authors, without undue reservation.

## Abbreviations

ATM, African traditional medicine; BUMETHA, Bushenyi medical and traditional healers; Association; IFC, informant consensus factor; TMP, traditional medicine practitioner; WHO, world health organization.

## References

[B1] Adia M. M. Anywar G. Byamukama R. Kamatenesi-Mugisha M. Sekagya Y. Kakudidi E. K. (2014). Medicinal plants used in malaria treatment by Prometra herbalists in Uganda. J. Ethnopharmacol. 155 (1), 580–588. 10.1016/j.jep.2014.05.060 24928824

[B2] Akwongo B. Katuura E. Nsubuga A. M. Tugume P. Andama M. Anywar G. (2022). Ethnobotanical study of medicinal plants utilized in the management of candidiasis in Northern Uganda. Trop. Med. Health 50 (1), 78–22. 10.1186/s41182-022-00471-y 36242066 PMC9569084

[B3] Alebie G. Urga B. Worku A. (2017). Systematic review on traditional medicinal plants used for the treatment of malaria in Ethiopia: trends and perspectives. Malar. J. 16, 307–313. 10.1186/s12936-017-1953-2 28764723 PMC5540187

[B4] Amri E. Kisangau D. P. (2012). Ethnomedicinal study of plants used in villages around Kimboza forest reserve in Morogoro, Tanzania. J. Ethnobiol. ethnomedicine 8 (1), 1–9. 10.1186/1746-4269-8-1 PMC3268735 22221935

[B5] Asiimwe S. Kamatenesi-Mugisha M. Namutebi A. Borg-Karlsson A.-K. Musiimenta P. (2013). Ethnobotanical study of nutri-medicinal plants used for the management of HIV/AIDS opportunistic ailments among the local communities of western Uganda. J. Ethnopharmacol. 150 (2), 639–648. 10.1016/j.jep.2013.09.017 24076461

[B6] Asiimwe S. Namukobe J. Byamukama R. Imalingat B. (2021). Ethnobotanical survey of medicinal plant species used by communities around Mabira and Mpanga Central Forest Reserves, Uganda. Trop. Med. Health 49 (1), 52. 10.1186/s41182-021-00341-z 34187581 PMC8243914

[B7] Bajunirwe F. Bangsberg D. R. Sethi A. K. (2013). Alcohol use and HIV serostatus of partner predict high-risk sexual behavior among patients receiving antiretroviral therapy in South Western Uganda. BMC public health 13, 430. 10.1186/1471-2458-13-430 23641795 PMC3645971

[B8] Bajunirwe F. Haberer J. E. Boum Y. Hunt P. Mocello R. Martin J. N. (2014). Comparison of self-reported alcohol consumption to phosphatidylethanol measurement among HIV-infected patients initiating antiretroviral treatment in southwestern Uganda. PLoS One 9 (12), e113152. 10.1371/journal.pone.0113152 25436894 PMC4249861

[B9] Bansal P. Banerjee S. (2016). Effect of withinia somnifera and shilajit on alcohol addiction in mice. Pharmacogn. Mag. 12 (Suppl. 2), S121–S128. 10.4103/0973-1296.182170 27279696 PMC4883068

[B10] Bardach A. E. Alcaraz A. O. Ciapponi A. Garay O. U. Riviere A. P. Palacios A. (2019). Alcohol consumption’s attributable disease burden and cost-effectiveness of targeted public health interventions: a systematic review of mathematical models. BMC public health 19 (1), 1378. 10.1186/s12889-019-7771-4 31655600 PMC6815367

[B11] Bushenyi District Local Government (2014) Local action plan (LAP) 2014-2016 Bushenyi district.

[B12] Carai M. A. Agabio R. Bombardelli E. Bourov I. Gessa G. L. Lobina C. (2000). Potential use of medicinal plants in the treatment of alcoholism. Fitoterapia 71, S38–S42. 10.1016/s0367-326x(00)00178-7 10930711

[B13] Carpenter J. E. LaPrad D. Dayo Y. DeGrote S. Williamson K. (2018). An overview of pharmacotherapy options for alcohol use disorder. Fed. Pract. 35 (10), 48–58. 30766325 PMC6248154

[B14] Catherine K. Mugisha K. M. Bright W. Ogwang P. E. (2020) “Factors affecting the use of nutri-medicinal plants by pregnant women in kyeizooba,” in Bushenyi district western Uganda.

[B15] Collin J. Casswell S. (2016). Alcohol and the sustainable development goals. Lancet 387 (10038), 2582–2583. 10.1016/S0140-6736(16)30827-3 27353807

[B16] Corkery J. M. (2018a). Ibogaine as a treatment for substance misuse: potential benefits and practical dangers. Prog. Brain Res. 242, 217–257. 10.1016/bs.pbr.2018.08.005 30471681

[B17] Corkery J. M. (2018b). Ibogaine as a treatment for substance misuse: potential benefits and practical dangers. Prog. Brain Res. 242, 217–257. 10.1016/bs.pbr.2018.08.005 30471681

[B18] De C. J. (2019) Promoting herbal medicine in Uganda: traditional health practitioners and government working together.

[B19] Degonda M. Scheidegger P. (2012). Traditional healing in Uganda: a statistical analysis of treatments by a group of traditional healers. Afr. J. Health Sci. 20 (1-2), 50–55.

[B20] Diamond M. B. Dalal S. Adebamowo C. Guwatudde D. Laurence C. Ajayi I. O. (2018). Prevalence and risk factor for injury in sub-Saharan Africa: a multicountry study. Inj. Prev. J. Int. Soc. Child Adolesc. Inj. Prev. 24 (4), 272–278. 10.1136/injuryprev-2016-042254 29118002

[B21] Falkenhaug S. (2023) Ibogaine as a treatment for alcohol use disorder: a preclinical investigation. Open Access Te Herenga Waka-Victoria University of Wellington.

[B22] Faruque M. O. Uddin S. B. Barlow J. W. Hu S. Dong S. Cai Q. (2018). Quantitative ethnobotany of medicinal plants used by indigenous communities in the Bandarban District of Bangladesh. Front. Pharmacol. 9, 40. 10.3389/fphar.2018.00040 29467652 PMC5808248

[B23] Ferreira-Borges C. Rehm J. Dias S. Babor T. Parry C. D. (2016). The impact of alcohol consumption on African people in 2012: an analysis of burden of disease. Trop. Med. Int. Health 21 (1), 52–60. 10.1111/tmi.12618 26448195

[B24] Feyisa K. Yismaw M. B. Yehualaw A. Tafere C. Demsie D. G. Bahiru B. (2023). Medicinal plants traditionally used to treat human ailments in Ethiopia: a Systematic Review. Phytomedicine Plus 4, 100516. 10.1016/j.phyplu.2023.100516

[B25] Frye C. (2018) Microsoft Excel 2019 step by step. Microsoft Press.

[B26] Girma Z. Abdela G. Awas T. (2022). Ethnobotanical study of medicinal plant species in Nensebo District, south-eastern Ethiopia. Ethnobot. Res. Appl. 24, 1–25. 10.32859/era.24.28.1-25

[B27] Griswold M. G. Fullman N. Hawley C. Arian N. Zimsen S. R. Tymeson H. D. (2018). Alcohol use and burden for 195 countries and territories, 1990–2016: a systematic analysis for the Global Burden of Disease Study 2016. Lancet 392 (10152), 1015–1035. 10.1016/S0140-6736(18)31310-2 30146330 PMC6148333

[B28] Gumisiriza H. Birungi G. Olet E. A. Sesaazi C. D. (2019). Medicinal plant species used by local communities around queen elizabeth national park, maramagambo central forest reserve and ihimbo central forest reserve, south western Uganda. J. Ethnopharmacol. 239, 111926. 10.1016/j.jep.2019.111926 31067488

[B29] Gumisiriza H. Olet E. A. Mukasa P. Lejju J. B. Omara T. (2023). Ethnomedicinal plants used for malaria treatment in Rukungiri District, Western Uganda. Trop. Med. Health 51 (1), 49. 10.1186/s41182-023-00541-9 37644587 PMC10466780

[B30] Hamill F. Apio S. Mubiru N. Mosango M. Bukenya-Ziraba R. Maganyi O. (2000). Traditional herbal drugs of southern Uganda, I. J. Ethnopharmacol. 70 (3), 281–300. 10.1016/s0378-8741(00)00180-x 10837990

[B31] Hamilton A. C. (2004). Medicinal plants, conservation and livelihoods. Biodivers. Conservation 13, 1477–1517. 10.1023/b:bioc.0000021333.23413.42

[B32] Im P. K. Wright N. Yang L. Chan K. H. Chen Y. Guo Y. (2023). Alcohol consumption and risks of more than 200 diseases in Chinese men. Nat. Med. 29, 1476–1486. 10.1038/s41591-023-02383-8 37291211 PMC10287564

[B33] Ipni (2012) The international plant names index. Kew: The Royal Botanic Gardens.

[B34] Kabwama S. N. Ndyanabangi S. Mutungi G. Wesonga R. Bahendeka S. K. Guwatudde D. (2016). Alcohol use among adults in Uganda: findings from the countrywide non-communicable diseases risk factor cross-sectional survey. Glob. health action 9 (1), 31302. 10.3402/gha.v9.31302 27491961 PMC4974493

[B35] Kamagaju L. Bizuru E. Minani V. Morandini R. Stévigny C. Ghanem G. (2013). An ethnobotanical survey of medicinal plants used in Rwanda for voluntary depigmentation. J. Ethnopharmacol. 150 (2), 708–717. 10.1016/j.jep.2013.09.031 24095698

[B36] Kamatenesi M. M. Acipa A. Oryem-Origa H. (2011). Medicinal plants of otwal and ngai sub counties in oyam district, northern Uganda. J. Ethnobiol. Ethnomedicine 7, 7–14. 10.1186/1746-4269-7-7 PMC3029220 21241484

[B37] Kamatenesi-Mugisha M. Oryem-Origa H. (2007). Medicinal plants used to induce labour during childbirth in western Uganda. J. Ethnopharmacol. 109 (1), 1–9. 10.1016/j.jep.2006.06.011 16901666

[B38] Lee M.-H. Kwak J. H. Jeon G. Lee J.-W. Seo J.-H. Lee H.-S. (2014). Red ginseng relieves the effects of alcohol consumption and hangover symptoms in healthy men: a randomized crossover study. Food and Funct. 5 (3), 528–534. 10.1039/c3fo60481k 24458173

[B39] Liu Q. Lawrence A. J. Liang J. H. (2011). Traditional Chinese medicine for treatment of alcoholism: from ancient to modern. Am. J. Chin. Med. 39 (1), 1–13. 10.1142/S0192415X11008609 21213394

[B40] Lu L. Liu Y. Zhu W. Shi J. Liu Y. Ling W. (2009). Traditional medicine in the treatment of drug addiction. Am. J. drug alcohol abuse 35 (1), 1–11. 10.1080/00952990802455469 19152199

[B41] Lukas S. E. Penetar D. Berko J. Vicens L. Palmer C. Mallya G. (2005). An extract of the Chinese herbal root kudzu reduces alcohol drinking by heavy drinkers in a naturalistic setting. Alcohol. Clin. Exp. Res. 29 (5), 756–762. 10.1097/01.alc.0000163499.64347.92 15897719

[B42] Lukas S. E. Penetar D. Su Z. Geaghan T. Maywalt M. Tracy M. (2013). A standardized kudzu extract (NPI-031) reduces alcohol consumption in nontreatment-seeking male heavy drinkers. Psychopharmacology 226, 65–73. 10.1007/s00213-012-2884-9 23070022 PMC3562758

[B43] Lutoti S. Kaggwa B. Kamba P. F. Mukonzo J. Sesaazi C. D. Katuura E. (2023). Ethnobotanical survey of medicinal plants used in breast cancer treatment by traditional health practitioners in Central Uganda. J. Multidiscip. Healthc. 16, 635–651. 10.2147/JMDH.S387256 36919184 PMC10008314

[B44] Maling S. Kabakyenga J. Muchunguzi C. Olet E. A. Alele P. E. (2023). Treatment outcomes of alcohol use disorder by traditional medicine practitioners using plant derivatives in southwestern Uganda: findings from in-depth interviews. Front. Psychiatry 14, 1185108. 10.3389/fpsyt.2023.1185108 37720895 PMC10502213

[B45] Martin G. J. (2010) Ethnobotany: a methods manual. Routledge.

[B46] Mbatudde M. Mucunguzi P. Lye K. A. (2007). Phenology of Asteraceae in selected districts of central Uganda. Afr. J. Ecol. 45, 67–72. 10.1111/j.1365-2028.2007.00860.x

[B47] Moges A. Moges Y. (2019). Ethiopian common medicinal plants: their parts and uses in traditional medicine-ecology and quality control. Plant science-structure, Anat. physiology plants Cult. vivo vitro 21. 10.5772/intechopen.86202

[B48] Mongalo N. I. Raletsena M. V. (2022). An inventory of South African medicinal plants used in the management of sexually transmitted and related opportunistic infections: an appraisal and some scientific evidence (1990-2020). Plants (Basel) 11 (23), 3241. 10.3390/plants11233241 36501281 PMC9738887

[B49] Morse J. M. (2015) Analytic strategies and sample size. Los Angeles, CA: SAGE Publications Sage CA. 10.1177/1049732315602867 26355022

[B50] Mukazayire M.-J. Minani V. Ruffo C. K. Bizuru E. Stévigny C. Duez P. (2011). Traditional phytotherapy remedies used in Southern Rwanda for the treatment of liver diseases. J. Ethnopharmacol. 138 (2), 415–431. 10.1016/j.jep.2011.09.025 21963560

[B51] Nabifo S. C. Tsai A. C. Bajunirwe F. (2021). HIV-related stigma and its association with HIV transmission risk behaviors among boda boda motorcyclists in Mbarara Municipality, southwestern Uganda. Int. J. STD AIDS 32 (9), 791–798. 10.1177/0956462420987760 33769905

[B52] Namukobe J. Kasenene J. M. Kiremire B. T. Byamukama R. Kamatenesi-Mugisha M. Krief S. (2011). Traditional plants used for medicinal purposes by local communities around the Northern sector of Kibale National Park, Uganda. J. Ethnopharmacol. 136 (1), 236–245. 10.1016/j.jep.2011.04.044 21550390

[B53] Nankaya J. Gichuki N. Lukhoba C. Balslev H. (2019a). Medicinal plants of the Maasai of Kenya: a review. Plants (Basel) 9 (1), 44. 10.3390/plants9010044 31892133 PMC7020225

[B54] Nankaya J. Gichuki N. Lukhoba C. Balslev H. (2019b). Medicinal plants of the Maasai of Kenya: a review. Plants 9 (1), 44. 10.3390/plants9010044 31892133 PMC7020225

[B55] Ohurira T. Iyer H. S. Wagman J. A. Hahn J. A. Bajunirwe F. (2023). Proximity to alcohol sellers and dose response relationship between alcohol consumption with intimate partner violence in rural southwestern Uganda. J. Interpers. violence 38 (1-2), 1040–1059. 10.1177/08862605221086648 35438584

[B56] Ojelel S. Mucunguzi P. Katuura E. Kakudidi E. K. Namaganda M. Kalema J. (2019). Wild edible plants used by communities in and around selected forest reserves of Teso-Karamoja region, Uganda. J. Ethnobiol. ethnomedicine 15, 3–14. 10.1186/s13002-018-0278-8 PMC6327394 30626418

[B57] Okot D. F. Anywar G. Namukobe J. Byamukama R. (2020). Medicinal plants species used by herbalists in the treatment of snakebite envenomation in Uganda. Trop. Med. Health 48 (1), 44. 10.1186/s41182-020-00229-4 32518500 PMC7273665

[B58] Opio D. Andama E. Kureh G. (2018). Ethnobotanical survey of antimalarial plants in areas of: abukamola, angeta, oculokori and omarari of alebtong district in northern Uganda. Eur. J. Med. plants 21 (4), 1–14. 10.9734/ejmp/2017/38043

[B59] Oryema C. Rutaro K. Oyet S. W. Malinga G. M. (2021). Ethnobotanical plants used in the management of symptoms of tuberculosis in rural Uganda. Trop. Med. Health 49 (1), 92. 10.1186/s41182-021-00384-2 34809718 PMC8607616

[B60] Parker C. Scott S. Geddes A. (2019) Snowball sampling. SAGE research methods foundations.

[B61] Petelka J. Plagg B. Säumel I. Zerbe S. (2020). Traditional medicinal plants in South Tyrol (northern Italy, southern Alps): biodiversity and use. J. Ethnobiol. Ethnomedicine 16 (1), 74. 10.1186/s13002-020-00419-8 PMC7690129 33243238

[B62] Primah K. (2023). Factors influencing the use of traditional medicine during labour among women attending maternity ward at ishaka adventist hospital, Bushenyi district. IAA J. Biol. Sci. 10 (1), 18–37.

[B63] Rezvani A. H. Overstreet D. Yang Y. Clark Jr E. (1999). Attenuation of alcohol intake by extract of *Hypericum perforatum* (St John's wort) in two different strains of alcohol-preferring rats. Alcohol Alcohol. 34 (5), 699–705. 10.1093/alcalc/34.5.699 10528812

[B64] Santos G. M. Emenyonu N. I. Bajunirwe F. Rain Mocello A. Martin J. N. Vittinghoff E. (2014). Self-reported alcohol abstinence associated with ART initiation among HIV-infected persons in rural Uganda. Drug alcohol dependence 134, 151–157. 10.1016/j.drugalcdep.2013.09.025 24169501 PMC3885244

[B65] Schultz F. Anywar G. Wack B. Quave C. L. Garbe L.-A. (2020). Ethnobotanical study of selected medicinal plants traditionally used in the rural Greater Mpigi region of Uganda. J. Ethnopharmacol. 256, 112742. 10.1016/j.jep.2020.112742 32224196

[B66] Singh L. Joshi T. Tewari D. Echeverría J. Mocan A. Sah A. N. (2020). Ethnopharmacological applications targeting alcohol abuse: overview and outlook. Front. Pharmacol. 10, 1593. 10.3389/fphar.2019.01593 32116660 PMC7034411

[B67] Skinner M. D. Lahmek P. Pham H. Aubin H.-J. (2014). Disulfiram efficacy in the treatment of alcohol dependence: a meta-analysis. PloS one 9 (2), e87366. 10.1371/journal.pone.0087366 24520330 PMC3919718

[B68] Ssegawa P. Kasenene J. M. (2007). Medicinal plant diversity and uses in the Sango bay area, Southern Uganda. J. Ethnopharmacol. 113 (3), 521–540. 10.1016/j.jep.2007.07.014 17720338

[B69] Ssenku J. E. Okurut S. A. Namuli A. Kudamba A. Tugume P. Matovu P. (2022). Medicinal plant use, conservation, and the associated traditional knowledge in rural communities in Eastern Uganda. Trop. Med. Health 50 (1), 39. 10.1186/s41182-022-00428-1 35668541 PMC9168352

[B70] Tabuti J. R. Obakiro S. B. Nabatanzi A. Anywar G. Nambejja C. Mutyaba M. R. (2023). Medicinal plants used for treatment of malaria by indigenous communities of Tororo District, Eastern Uganda. Trop. Med. Health 51 (1), 34. 10.1186/s41182-023-00526-8 37303066 PMC10258082

[B71] Tardío J. Pardo-de-Santayana M. (2008). Cultural importance indices: a comparative analysis based on the useful wild plants of Southern Cantabria (Northern Spain). Econ. Bot. 62, 24–39. 10.1007/s12231-007-9004-5

[B72] Tefera B. N. Kim Y.-D. (2019). Ethnobotanical study of medicinal plants in the hawassa zuria district, sidama zone, southern Ethiopia. J. Ethnobiol. ethnomedicine 15, 25–21. 10.1186/s13002-019-0302-7 PMC6534827 31126296

[B73] Tomczyk M. Zovko-Končić M. Chrostek L. (2012). Phytotherapy of alcoholism. Nat. Product. Commun. 7 (2), 1934578X1200700–280. 10.1177/1934578x1200700243 22474979

[B74] Tugume P. Kakudidi E. K. Buyinza M. Namaalwa J. Kamatenesi M. Mucunguzi P. (2016). Ethnobotanical survey of medicinal plant species used by communities around Mabira Central Forest Reserve, Uganda. J. Ethnobiol. Ethnomedicine 12 (1), 5. 10.1186/s13002-015-0077-4 PMC4712608 26762159

[B75] Tumwesigye O. (1996). Bumetha Rukararwe: integrating modern and traditional health care in southwest Uganda. J. Altern. Complementary Med. 2 (3), 373–376. 10.1089/acm.1996.2.373 9395672

[B76] UBOS (2014) Uganda national population and housing census 2014. Uganda Bureau of Statistics: Kampala-Uganda.

[B77] WHO (2001) Legal status of traditional medicine and complementary. Geneva: World Health Organisation. Report No.: 9241545488.

[B78] WHO (2013a) WHO traditional medicine strategy: 2014-2023. Geneva: World Health Organization. Report No.: 9241506091.

[B79] WHO (2013b) WHO traditional medicine strategy: 2014-2023. World Health Organization.

[B80] WHO (2019a) Global status report on alcohol and health 2018. World Health Organization.

[B81] WHO (2019b) Global status report on alcohol and health 2018. World Health Organization. Report No.: 9241565632.

[B82] Xu B. J. Zheng Y. N. Sung C. K. (2005). Natural medicines for alcoholism treatment: a review. Drug Alcohol Rev. 24 (6), 525–536. 10.1080/09595230500293795 16361209

